# LncRNA small nucleolar RNA host gene 5 inhibits trophoblast autophagy in preeclampsia by targeting microRNA-31-5p and promoting the transcription of secreted protein acidic and rich in cysteine

**DOI:** 10.1080/21655979.2022.2040873

**Published:** 2022-03-08

**Authors:** Lei Yang, Chao Liu, Chao Zhang, Ruotian Shang, Yichen Zhang, Shiyuan Wu, Yan Long

**Affiliations:** Department of Gynecology & Obstetrics, Beijing Friendship Hospital, Capital Medical University, Beijing, Xicheng, China

**Keywords:** Long non-coding RNA SNHG5, microRNA-31-5p, autophagy, preeclampsia, SPARC, trophoblast

## Abstract

Preeclampsia (PE) is a pregnancy-related complication. Dysregulation of long non-coding RNAs (lncRNAs) contributes to the pathogenesis of PE. The current study sought to investigate the effect of lncRNA small nucleolar RNA host gene 5 (SNHG5) on trophoblast autophagy in PE. A PE mouse model was established, followed by detection of parameters such as blood pressure, proteinuria, triglycerides, total cholesterol, low-density lipoprotein, and high-density lipoprotein, observation of alterations of mouse placenta and kidney, and detection of B-cell chronic lymphocytic leukemia/lymphoma-2, Bcl-2-associated X protein, and SNHG5 expression patterns. The expressions of LC3, Beclin-1, and p62 in the placenta of PE mice were detected. Moreover, the SNHG5 expression was downregulated in the established HTR-8/SVneo trophoblast model, followed by evaluation of cell proliferation, apoptosis, and autophagy. After combination treatment with 3-MA (an autophagy inhibitor) and si-SNHG5, the behaviors of HTR-8/SVneo cells were observed. The binding relations between SNHG5 and miR-31-5p, and miR-31-5p and SPARC were verified. The expressions of miR-31-5p and SPARC in the placenta of mice and trophoblasts were determined. Our results demonstrated a poor expression of lncRNA SNHG5 in PE mice. SNHG5 overexpression reduced the PE phenotype and tissue damage in mice. SNHG5 silencing reduced the proliferation, migration, and invasion of trophoblasts, but elevated apoptosis and autophagy. SNHG5 sponged miR-31-5p to promote SPARC transcription. Additionally, miR-31-5p knockdown or 3-MA treatment reverted the stimulative effect of SNHG5 silencing on trophoblast autophagy. Collectively, our study demonstrated that lncRNA SNHG5 alleviated the PE phenotype and inhibited trophoblast autophagy by sponging miR-31-5p and promoting SPARC transcription.

## Graphical abstract

**Figure:**
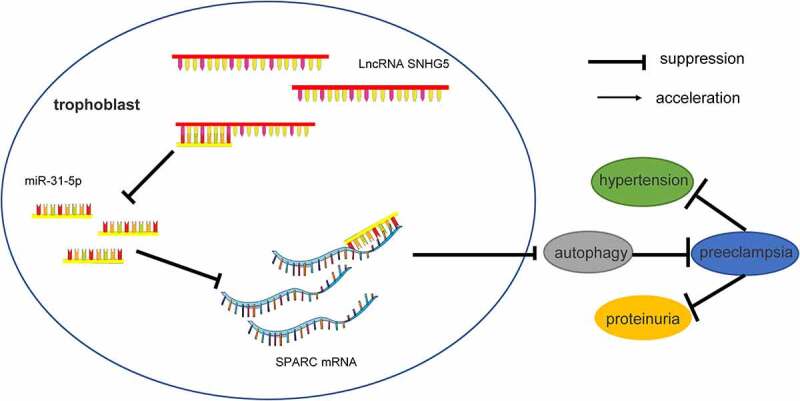

## Introduction

Preeclampsia (PE), a pregnancy-specific syndrome, is responsible for the high mortality rate during the maternal and perinatal period and is associated with factors such as systemic organ impairments [1], hematological and neural comorbidities, and cardiovascular events in gestational women, prominently in developing regions [2]. The risk factors of PE include endothelial dysfunction, kidney disorders, maternal obesity, hypertension, insufficient intrauterine fetal development, and an elaborate family history of PE [3]. Essentially, PE is characteristic of the unhealthy placenta, elevated levels of proteinuria with hypertension, as well as trophoblast autophagy [4]. A regimen with the proper administration of folic acid, statin, and aspirin has a preventive effect in PE [5]. However, the efficacy of these drugs has been limited to a few PE patients [6]. In this context, the development of novel therapeutic strategies for PE is warranted.

Long non-coding RNAs (LncRNAs) are abnormally expressed in PE and function as modulators of placenta structure, endothelial function, and uteroplacental circulation [7]. Furthermore, lncRNAs can mediate trophoblast expansion, development, mobility, and differentiation to facilitate PE progression [8]. LncRNA small nucleolar RNA host gene 5 (SNHG5) has been classified as a putative biomarker for disease treatment with an aberrant expression in various human diseases and participation in the regulation of cell reproduction, viability, self-renewal, and death [9,10]. Consistently, an existing study determined that lncRNA SNHG5 is poorly expressed in PE, and can regulate trophoblast cell proliferation, invasion, and migration [11]. However, the effect of lncRNA SNHG5 on trophoblast autophagy in PE remains elusive. Therefore, we selected lncRNA SNHG5 as the research target to determine whether SNHG5 is involved in the development of PE by regulating trophoblast autophagy.

Notably, lncRNA SNHG5 elicits its function via interaction of the competing endogenous RNA (ceRNA) to sponge its downstream microRNAs (miRNAs) [12]. Recently, The role of ceRNA crosstalk has gained significant attention in PE pathological progression [13]. As a series of gene expression modulators, several miRNAs are implicated in PE due to their ability to mediate trophoblast viability and apoptosis, immune reactions, and placenta hypoxia [14]. Therefore, we predicted the downstream miRNAs of SNHG5 through the RNA22 database, among which miR-31-5p presented with a prominent expression in the placental tissues and trophoblasts of PE patients [15]. Additionally, miR-31-5p overexpression can serve as an indicator of increased proteinuria and hypertension in PE patients [16]. miR-31-5p can weaken trophoblast functions and subsequently exacerbate PE [15]. Furthermore, the TargetScan database predicted that miR-31-5p can target secreted protein acidic and rich in cysteine (SPARC). SPARC is a matricellular molecule that functions as a modulator of interactions between cells and their surrounding extracellular matrix. An existing study identified a considerably lower SPARC expression in PE placenta relative to the normal placenta in late pregnancy, thus eliciting its function as a terminal biomarker for PE [17]. Manipulation of the SPARC expression can reduce the invasion of human trophoblast cells [18]. Therefore, we speculated that lncRNA SNHG5 can target SPARC transcription by competitively binding to miR-31-5p, thereby functioning as a modulator of trophoblast autophagy in PE. The current study sought to determine the underlying mechanism of the lncRNA SNHG5/miR-31-5p/SPARC axis in trophoblast autophagy in PE to provide an insight into the clinical management of PE.

## Materials and methods

### Ethics statement

This study was conducted with the approval of the ethics committee of Beijing Friendship Hospital, Capital Medical University (18–2032). The protocol was also conducted in compliance with the *Guidelines for the Care and Use of Laboratory Animals* provisions of administration and usage of laboratory animals [19]. Adequate measures were taken to minimize both the number of animals and their suffering.

### Mouse model establishment

A total of forty-eight female C57BL/6 J mice (8–10 weeks old) and 24 male C57BL/6 J mice (10–14 weeks old) (all from Slac Laboratory Animals Ltd, Shanghai, China, SYXK (Shanghai) 2017–0008) were housed in 12-h light/dark cycles at 23 ± 2°C and 55 ± 10% relative humidity with ad libitum access to food and water, and the mice were raised in a ratio of 2 females to 1 male in one cage to facilitate mating [20]. The identification of a white or faint yellow vaginal plug during the early hours of the following day was regarded as the 0.5 embryonic days. TLR9 agonist ODN1826 (5 mg/kg/once, Santa Ctuz Biotechnology, Inc, Santa Cruz, CA, USA) was injected intraperitoneally into the pregnant mice to induce preeclampsia (PE group, N = 36) on E7.5. The untreated pregnant mice were classified into the control group (N = 12). The mice in the PE + oe-SNHG5 group and PE + oe-NC group were injected with lentivirus-packaged plasmids pCDNA3.1-SNHG5 (SNHG5 overexpression) or pCDNA3.1-negative control (NC) (3 × 10^9^ PFU) via tail vein. On E17.5, the blood and urine were collected, after which the mice were euthanized with an injection containing 200 mg/kg sodium pentobarbital via the tail vein. Next, the kidney and placenta specimens were randomly selected from 6 mice in each group were and fixed in 4% paraformaldehyde for subsequent experimentation. The remaining 6 mice in each group were reserved for reverse transcription quantitative polymerase chain reaction (RT-qPCR) and Western blot assay.

### Blood pressure measurement

In compliance with an existing protocol [21], the diastolic and systolic blood pressure of conscious mice was measured based on the tail-cuff technique on the Visitech BP2000 system (Apex, NC, USA). Before formal evaluation, all mice underwent habitual measurement (from E4.5 to E6.5, 10 times per day). Next, the blood pressure levels throughout pregnancy were evaluated at E7.5, E9.5, E11.5, E13.5, and E17.5.

### Hematoxylin and eosin (H&E) staining

H&E staining was performed in strict accordance with an existing protocol [21]. The placenta and kidney specimens were fixed in 4% paraformaldehyde for at least a period of 12 h, paraffin-embedded, sliced into sections (4 μm), subjected to H&E staining, blocked using neutral resins, and then observed under a microscope.

### Assessment of proteinuria and blood lipid

The urine on E17.5 at gestation was withdrawn for assessment of proteinuria. Urine albumin was assessed in strict accordance with the provided protocol of the easy ll Protein Quantitative kits (DQ111-01; Beijing Transgen Biotech, Beijing, China). Urine was collected from each mouse for protein concentration determination based on the bicinchoninic acid (BCA) protein method. Optical density (OD) value at the excitation wavelength of 595 nm was determined using a microplate reader, with the bovine serum albumin (BSA, Sigma-Aldrich, Merck KGaA, Darmstadt, Germany) as the reference. The detection of proteinuria was conducted at least three times [22].

Blood sample was collected on E17.5 for blood lipid assessment using the UV-Vis spectrophotometry in strict accordance with the provided instructions of the blood lipid biochemical test kits for the following indices: total cholesterol (TC) (Bc1985; Beijing Solarbio Science & Technology Co., Ltd., Beijing China), triglyceride (TG) (BH017Z; Tellgen corporation, Shanghai, China), low-density lipoprotein (LDL) (BH019Z) and high-density lipoprotein (HDL) (BH018Z). The detection of blood lipid profile was conducted a minimum of three times [22].

### Immunohistochemistry

The light chain 3 (LC3) expression was detected using immunohistochemistry in compliance with an existing protocol [23]. The placental tissues were harvested, fixed with 4% paraformaldehyde, and embedded in paraffin. After conventional dewaxing and dehydration, the tissues were cultured with Anti-LC3 (at a dilution ratio of 1:200, ab48394, Abcam Inc., Cambridge, MA, USA) for 12 h at 4°C and then incubated with the horseradish peroxidase-coupled immunoglobulin G (IgG, at a dilution ratio of1:2000, ab6721, Abcam) for 30 min at room temperature, rinsed, stained with 3,3’-diaminobenzidine and then re-stained with hematoxylin for 2 min. After dehydration and sealing, the tissue sections were observed under a light microscope.

### Cell culture and transfection

As mentioned in an existing protocol [21,24], the trophoblast HTR-8/SVneo cells (ATCC, Manassas, VA, USA) were cultured in Roswell Park Memorial Institute-1640 medium (11,875–093; Thermo Fisher Scientific Inc., Waltham, MA, USA) containing a combination of 10% fetal bovine serum (Thermo Fisher Scientific) and 1% penicillin-streptomycin (15,140,148, Thermo Fisher Scientific) in a 37°C humidified incubator with 5% CO_2_. Next, 50 nM of small interfering (si)-SNHG5 or si-NC, and 100 nM of miR-31-5p inhibitor or inhibitor NC (all from Shanghai GenePharma Co., Ltd., Shanghai, China) were transfected into cells using Lipofectamine 2000 (Invitrogen Inc., Carlsbad, CA, USA). The cells were isolated after 48 h for subsequent experimentation. Additionally, 3-methyladenine (3-MA, MedChemExpress LLC, Monmouth Junction, NJ, USA), an autophagy inhibitor, was dissolved in the dimethyl sulfoxide (DMSO) solution. Then, 5 mM of 3-MA was supplemented into the medium, with the DMSO solution as a control. The transfected cells were pre-treated for 48 h for subsequent experimentation.

### Assessment of cell viability and proliferation

The Cell Counting Kit-8 (CCK-8, Beyotime, Shanghai, China) was employed for the assessment of HTR-8/SVneo cell viability and proliferation [11]. Briefly, 100 μL of HTR-8/SVneo cell medium (1 × 10^5^ cells/mL) was supplemented in 96-well plates and cultured at 37°C with 5% CO_2_. The CCK-8 solution was added into 96-well plates for 1.5 h-incubation regimens at 24, 36, 48, and 60 h, respectively. The OD value at the excitation wavelength of 450 nm was determined using a microplate reader (Bio-Rad 680, Hercules, CA, USA) to estimate cell proliferation.

### Transwell assays

Transwell assays were performed to test the migration and invasion abilities of HTR-8/SVneo cells [25]. For the cell migration test, the HTR-8/SVneo cells (1 × 10^6^) were added to the apical chamber of the Transwell containing serum-free RPMI-1640 medium for 15 min of incubation at 37°C. The basolateral chamber was supplemented with 700 μL of RPMI-1640 medium containing 10% fetal bovine serum (FBS) and the cells were cultured for 72 h. The Transwell plate was fixed using 70% ethanol. Next, each well was supplemented with 0.2% crystal violet to stain the membrane-crossing cells for subsequent observation and counting under an inverted microscope (Olympus, Tokyo, Japan).

For the cell invasion test, the HTR-8/SVneo trophoblasts (1 × 10^6^) were added to the apical chamber of the Transwell containing serum-free RPMI-1640 for incubation with Matrigel (Millipore Corp., Billerica, MA, USA) for 15 min at 37°C. The basolateral chamber was supplemented with 700 μL of RPMI-1640 containing 10% FBS and cells were cultured for 72 h. With the elimination of Matrigel from filtered cells passing through the apical chamber, the Transwell plate was fixed with 70% ethanol. Next, each well was supplemented with 0.2% crystal violet to stain the membrane-crossing cells, and the stained cells were observed and counted under an inverted microscope (Olympus).

### Flow cytometry

In compliance with an existing protocol [15], flow cytometry was conducted to detect HTR-8/SVneo apoptosis. The Annexin V-fluorescein isothiocyanate (FITC)/propidium iodide (PI) apoptosis assay kit (YEASEN Biotechnology Co. Ltd., Shanghai, China) was subsequently adopted. The HTR-8/SVneo cells were isolated, centrifuged, transfected for 45 h, and resuspended with the binding buffer. The cell suspension was stained with Annexin V-FITC/PI. The apoptosis rate was determined using the Attune™ NxT flow cytometer (Invitrogen, Thermo Fisher Scientific).

### RFP-GFP-LC3 tandem fluorescence assay

Autophagic flux was detected in strict accordance with an existing protocol [26]. Trophoblasts were seeded into 12-well plates, treated with rapamycin (100 nM, Millipore Sigma) for 3 h, and co-cultured with the RFP-GFP-LC3 adenovirus (constructed by Genomeditech Co., Ltd., Shanghai, China) for 24 h. Next, the experimental cells were transfected with si-SNHG5, si-SNHG5 + 3 MA, si-SNHG5 + miR-31-5p inhibitor, or corresponding controls respectively for 24 h. The degree of autophagic flux was evaluated under a fluorescence microscope.

### RT-qPCR

Bax, Bcl-2, lncRNA SNHG5, miR-31-5p, and SPARC expression patterns in the mouse placental tissues or HTR-8/SVneo cells were determined using RT-qPCR. The total RNA content was extracted from the placenta tissues or HTR-8/SVneo cells in strict accordance with the provided instructions of the TRIzol reagent (Invitrogen). Initially, mRNA was reverse transcribed into cDNA using the PrimeScript RT Master Mix (Takara, Dalian, China). Next, miRNA was reverse transcribed into cDNA using the PrimeScript ll 1^st^ Strand cDNA Synthesis Kit. Real-time PCR was conducted using the SYBR Premix Ex Taq kit (Thermo Fischer Scientific) on the ABI 7500 system (Applied Biosystems, Foster City, CA, USA) [11], with glyceraldehyde-3-phosphate dehydrogenase (GAPDH) and U6 as the internal reference. The gene relative expression was estimated based on the 2^−ΔΔCt^ method. The RT-qPCR primers were seen in Table 1.

**Table 1.** Primer sequence of RT-qPCR

	Forward Primer (5’-3’)	Reverse Primer (5’-3’)	PCR Product size
Bax	CTGGATCCAAGACCAGGGTG	GTGAGGACTCCAGCCACAAA	179 bp
Bcl-2	GAACTGGGGGAGGATTGTGG	GCATGCTGGGGCCATATAGT	211 bp
SNHG5	AGCTGCATCGCCTTTACCTT	TGCAGCCTTTTGTGCTGTTC	184 bp
miR-31-5p	GCCGAGAGGCAAGATGCTGGCA	CTCAACTGGTGTCGTGGA	122 bp
SPARC	GGAAGAAACTGTGGCAGAGG	ATTGCTGCACACCTTCTCAA	166 bp
GAPDH	AATGGATTTGGACGCATTGGT	TTTGCACTGGTACGTGTTGAT	180 bp
U6	CTCGCTTCGGCAGCACA	AACGCTTCACGAATTTGCGT	107 bp

Note: RT-qPCR, reverse transcription-quantitative polymerase chain reaction; Bcl-2, B-cell lymphoma-2; BAX, Bcl-2-associated X; SNHG5, small nucleolar RNA host gene 5; miR, microRNA; SPARC, secreted protein acidic and rich in cysteine; GAPDH, glyceraldehyde-3-phosphate dehydrogenase

### Western blot analysis

The LC3, Beclin 1, and p62 protein levels were determined using Western blot analysis [24]. The cells were lysed using the enhanced radio-immunoprecipitation assay lysis solution (Invitrogen) containing several protease inhibitors to extract the total protein content, followed by determination of protein concentration using the BCA protein assay kit (Thermo Fisher Scientific). The proteins (30 μg/well) were separated through sodium dodecyl sulfate-polyacrylamide gel electrophoresis and then transferred onto polyvinylidene fluoride membranes (Thermo Fischer Scientific), followed by membrane blockade using 4% BSA for 1 h at 4°C. After a rinse, the membranes were incubated with the primary antibodies (all from Abcam): Anti-LC3 (at a dilution ratio of 1: 2000, ab48394), Anti-Beclin1 (at a dilution ratio of 1: 100, ab62557), Anti-p62 (at a dilution ratio of 1: 200, ab91526) and Anti-beta Actin (at a dilution ratio of 1: 1000, ab8227) at 4°C overnight. Next, the membranes were cultivated with horseradish peroxidase-coupled IgG (at a dilution ratio of 1: 2000, ab6721, Abcam) at 24°C for 1 h. Blots were detected using the Pierce ECL Plus Substrate (Thermo Fisher Scientific). Digital images of the observations were obtained using the MiVnt imaging analysis system (Bio-Rad). The bands were analyzed using the ImageJ software, and the band intensity was standardized to β-actin.

### Fractionation of nuclear and cytoplasmic RNA

The fractionation of nuclear and cytoplasmic RNA was conducted in strict accordance with an existing protocol [27]. PARIS Kit (AM1921, Thermo Fisher Scientific) was employed for subcellular isolation. The HTR-8/SVneo trophoblasts were isolated, rinsed with PBS, placed on ice, and resuspended using 100 μL of pre-cooled cell fractionation buffer. Next, the cells were incubated on ice for 10 min and then centrifuged, followed by extraction of the cytoplasm, with the remaining sections as nuclear granules. The nuclear granules were rinsed with pre-cooled cell fractionation buffer and lysed with the cytolytic buffer. The lysate was mixed with an equivalent amount of 2 × lysis/binding solution and supplemented with an equivalent volume of 100% ethanol. The sample mixture was extracted through a cartridge, rinsed several times with the washing solution, and treated with an eluent to isolate the RNA content. Reverse transcription was performed using the M-MLV kit (No. 28,025,013; Thermo Fisher Scientific). The SNHG5 expression in the nucleus and cytoplasm was detected by RT-qPCR, with GAPDH and U6 as controls.

### Bioinformatics

Subcellular localization of SNHG5 was predicted via the LncATLAS (http://lncatlas.crg.eu/) [28]. The binding sites between SNHG5 and miR-31-5p and between miR-31-5p and SPARC were predicted through a combination of RNA22 (https://cm.jefferson.edu/rna22/Interactive/) [29] and Targetscan (http://www.targetscan.org/) websites [30].

### Dual-luciferase reporter gene assay

The binding relationships between lncRNA SNHG5 and miR-31-5p, and miR-31-5p and SPARC were verified using dual-luciferase reporter gene assay [31]. The wild type (WT) and mutant type (MUT) of SNHG5 and SPARC fragments containing the binding sites of miR-31-5p were constructed into the pMIR-reporter vector (Beijing Huayueyang Biotechnology, Beijing, China). The constructed dual-luciferase reporter plasmids were co-transfected with the mimic-NC or miR-31-5p-mimic into the HTR-8/SVneo trophoblasts. After 48 h, the cells were isolated, lysed, and evaluated using the luciferase assay system (K801-200; Biovision, Mountain View, CA, USA) for analysis of the luciferase activity.

### RNA pull-down assay

RNA pull-down assay was performed in compliance with an existing protocol [32]. The HTR-8/SVneo trophoblasts in the logarithmic growth phase were isolated, centrifuged at 4°C for 5 min, and then mixed with Pierce IP Lysis Buffer (Thermo Fisher Scientific). After 30 min of lysis on ice, the cells were centrifuged at 4°C for 30 min to isolate the supernatant, which was then subject to RNA pull-down assay using the Pierce Magnetic RNA Protein pull-down kit in strict accordance with the provided protocol. Biotinylated miR-31-5p and NC probes were dissolved in washing/binding buffer and incubated with the streptavidin-coupled magnetic beads for 2 h at 4°C. The probes were added to the cell lysate for 2 h for complete elution of the RNA complexes bound to the magnetic beads. Moreover, RT-qPCR was conducted to measure the RNA expression.

### Statistical analysis

SPSS 21.0 software (IBM Corp. Armonk, NY, USA) was used for data analysis while the GraphPad Prism 8.0 software (GraphPad Software Inc., San Diego, CA, USA) was used for graphing. The experimental data were expressed as cases and percentages, and the chi-square test was adopted for comparisons between groups. The measurement data were presented as mean ± standard deviation. All data were in conformity with normality distribution and homogeneity test of variance. The *t*-test was adopted for comparison analysis between two groups and one-way or two-way analysis of variance (ANOVA) was adopted for comparison analysis among multiple groups, and the Tukey’s multiple comparisons test was adopted for posttest of data. The *p* value was attained using a two-tailed test and in all statistical references, a value of *p* < 0.05 was indicative of a significant difference.

## Results

This study sought to investigate the mechanism of abnormal trophoblast autophagy mediated by lncRNA SNHG5/miR-31-5p in the pathogenesis of PE. In this study, we initially analyzed the expression pattern of SNHG5 in PE mice and then analyzed the relationship between SNHG5 and mouse PE phenotype and trophoblast autophagy. Next, we transfected si-SNHG5 into the HTR8/SVneo cells to study the effects of SNHG5 on HTR8/SVneo cell proliferation, migration, invasion, apoptosis, and autophagy. Thereafter, we determined and verified the downstream mechanism of SNHG5. Our results revealed that SNHG5 could competitively bind to miR-31-5p through the ceRNA mechanism to induce SPARC transcription. Finally, the rescue experiment was conducted by inhibiting miR-31-5p.

### SNHG5 is poorly expressed in PE mice, and SNHG5 overexpression alleviates PE phenotype and tissue injury in PE mice

**Figure 1.**
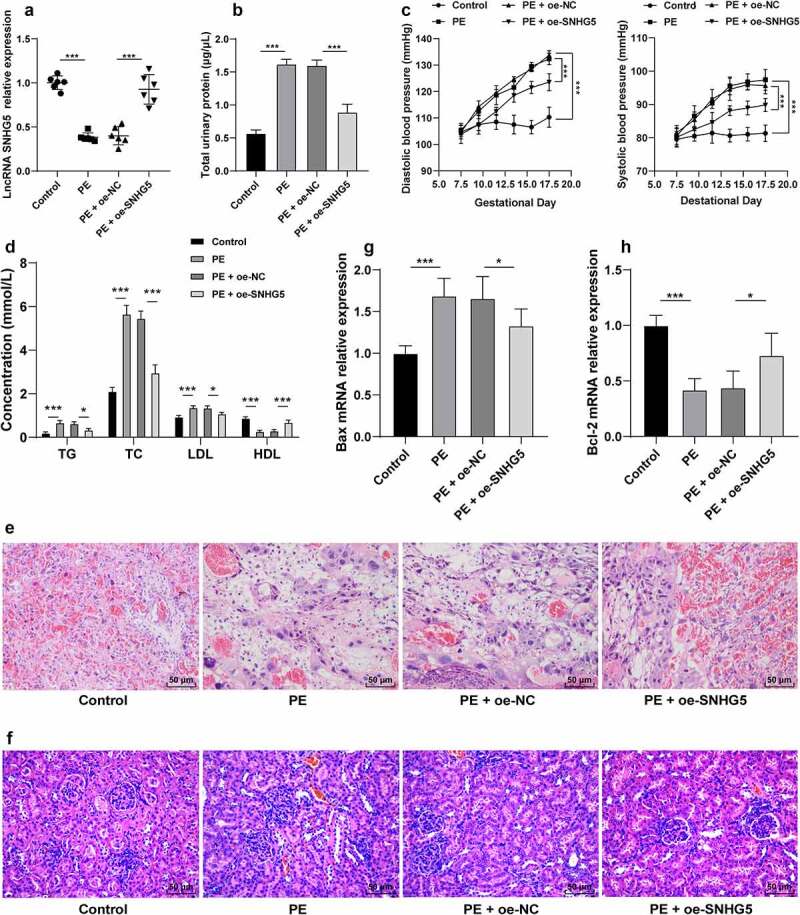
SNHG5 is poorly expressed in PE mice, and SNHG5 overexpression alleviates PE phenotype and tissue injury in PE mice. The PE mouse model was established, with healthy mice as the control; and pCDNA3.1-SNHG5 was injected into mice via tail vein, with pCDNA3.1-NC injection as the control. (A) SNHG5 expression pattern in mouse placental tissues was detected by RT-qPCR. (B) proteinuria was extracted from mice using the easy ll Protein Quantitative kits. (C) blood pressure was determined by the tail-cuff technique. (D) levels of TG, TC, LDL, and HDL were measured using the lipid biochemical assay kits. E and F, placenta tissue (e) and kidney tissue (f) were observed by H&E staining. (G) and (H) mRNA expression patterns of Bax (g) and Bcl-2 (h) were assessed by RT-qPCR. The results were presented as mean ± standard deviation. One-way ANOVA was used to analyze the data in panels (A), (B), (G), and (H), and two-way ANOVA was used to analyze the data in panels (C) and (D). Tukey’s multiple comparisons test was applied for the post hoc test. * *p* < 0.05, ** *p* < 0.01, *** *p* < 0.001.

To investigate the role of SNHG5 in PE, the PE mouse model was established to examine the expression pattern of SNHG5, after which a poor SNHG5 expression pattern was identified in the PE mice (*p* < 0.05, Figure 1a). On E17.5, elevated proteinuria and blood pressure were determined in the PE mice (*p* < 0.05, Figure 1b-C). The levels of TG, TC, LDL, and HDL in PE mice were measured using the lipid biochemical assay kit, which revealed elevated TG, TC, and LDL levels in PE mice relative to the control mice, while the HDL level was lower in PE mice than the control mice (*p* < 0.05, Figure 1d). The placental and kidney tissue sections of mice were observed by H&E staining, which revealed the PE mouse placenta with hyperplasia, reduced vascularity in the chorionic villi, villous stroma edema hyperplasia, and fibrinoid necrosis; the kidney tissue presented with glomerular constriction, moderate swelling of the glomerular endothelial cells and thylakoid cells, and occlusion of glomerular capillaries (Figure 1e-F). RT-qPCR was conducted to detect the Bax and Bcl-2 expression patterns in the PE mice and revealed that Bax was elevated while Bcl-2 was weakened in the PE mice compared to the control mice (*p* < 0.05, Figure 1g-H). pCDNA-SNHG5 was injected into the experimental mice and it revealed that SNHG5 overexpression could alleviate PE phenotype and tissue injury in PE mice (*p* < 0.05, Figure 1a-H).

### SNHG5 overexpression suppresses autophagy in PE mice

**Figure 2.**
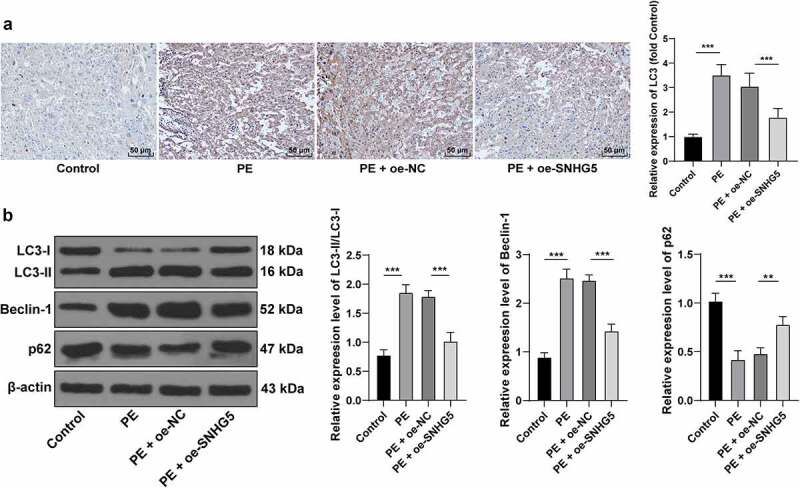
SNHG5 overexpression suppresses autophagy in PE mice. pCDNA3.1-SNHG5 was injected into mice via tail vein, with pCDNA3.1-NC injection as the control. (A) LC3 expression pattern was measured by immunohistochemistry. (B) levels of LC3, Beclin-1, and p62 were measured by Western blot analysis. The results were presented as mean ± standard deviation. One-way ANOVA was used to analyze the data in panels A and B. Tukey’s multiple comparisons test was applied for the post hoc test. ** *p* < 0.01, *** *p* < 0.001.

Autophagy is associated with vigorous development and pathophysiology of PE [21,33]. Therefore, the relationship between SNHG5 and autophagy in PE mice was subsequently investigated. Immunohistochemistry showed an elevated LC3 expression pattern in the PE group compared to the normal group, while it was decreased in the PE + oe-SNHG5 group (*p* < 0.05, Figure 2a). Autophagy was assessed with detection of the protein levels of LC3, Beclin-1, and p62, and the results presented with considerably high expression pattern of Beclin-1 in the PE mouse tissues, the relative expression pattern of LC3II/LC3l was increased, and p62 was poorly expressed, which were all reverted with SNHG5 overexpression (*p* < 0.05, Figure 2b). The aforementioned results indicated that autophagy had evidently increased in the PE mice, while SNHG5 overexpression inhibited autophagy in PE mice.

### SNHG5 silencing promotes trophoblast autophagy

**Figure 3.**
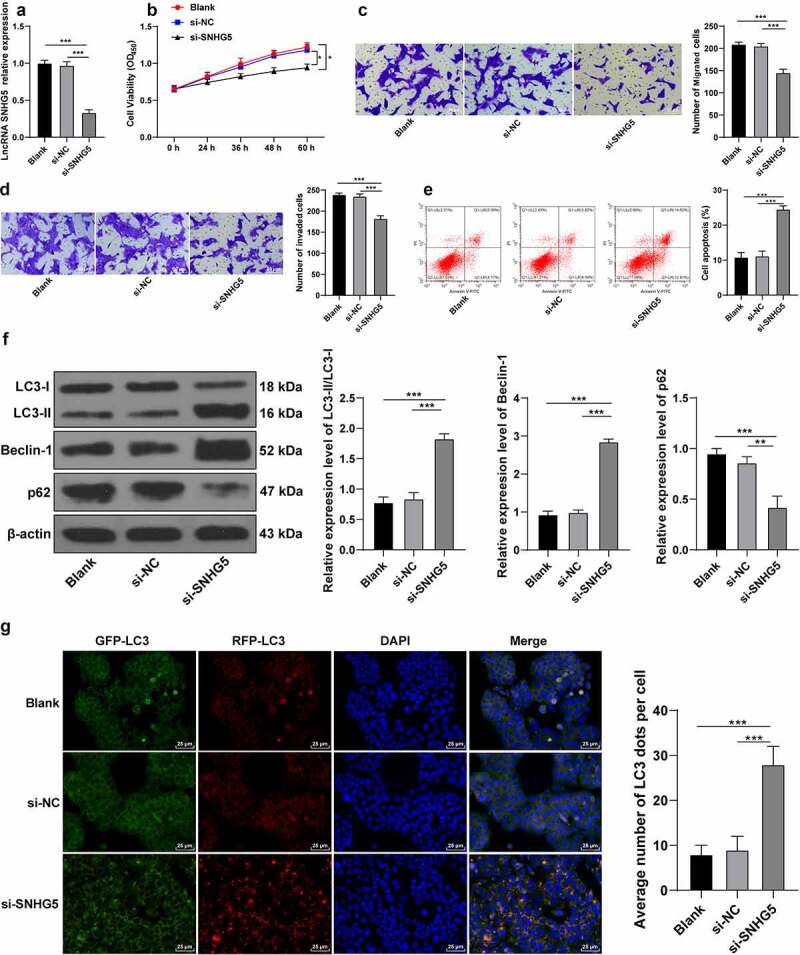
SNHG5 silencing promotes trophoblast autophagy. si-SNHG5 was transfected into HTR-8/SVneo cells, with si-NC transfection as the control. (A) si-SNHG5 transfection efficiency was detected by RT-qPCR. (B) trophoblast proliferation was determined by CCK-8 method. (C) and (D) cell migration (c) and invasion (d) were measured by Transwell assays. (E) trophoblast apoptosis was assessed by flow cytometry. (F) levels of LC3, Beclin-1, and p62 were measured by Western blot analysis. (G), trophoblast autophagic flux was analyzed by RFP-GFP-LC3 tandem fluorescence assay. The independent experiments were performed 3 times. The results were presented as mean ± standard deviation. One-way ANOVA was used to analyze the data in panels (A) (C) (D) (E) (F) and (G) and two-way ANOVA was used to analyze the data in panel (B). Tukey’s multiple comparisons test was applied for the post hoc test. ** *p* < 0.01, *** *p* < 0.001.

To investigate the critical role of SNHG5 in PE development, si-NC or si-SNHG5 was transfected into HTR-8/SVneo cells to subsequently downregulate the SNHG5 expression pattern in experimental cells (*p* < 0.05, Figure 3a). The CCK-8 protocol revealed that SNHG5 silencing impeded HTR-8/SVneo cell proliferation (*p* < 0.05, Figure 3b). Transwell assays revealed that SNHG5 silencing inhibited HTR-8/SVneo cell migration and invasion (*p* < 0.05, Figure 3c-D). Flow cytometry revealed that SNHG5 silencing elevated the degree of apoptosis (*p* < 0.05, Figure 3e). Western blot analysis demonstrated that SNHG5 silencing upregulated the relative expression pattern of LC3II/LC3l and Beclin-1 expression pattern, while it reduced the p62 expression pattern (*p* < 0.05, figure 3f). RFP-GFP-LC3 tandem fluorescence assay revealed that SNHG5 silencing increased the concentration of autophagosomes and autolysosomes in the HTR-8/SVneo cells (*p* < 0.05, Figure 3g). The preceding findings suggested that SNHG5 silencing exacerbated autophagy in trophoblasts (*p* < 0.05, figure 3f-G).

### 3-MA counteracts the promotive role of SNHG5 silencing in trophoblast autophagy

**Figure 4..**
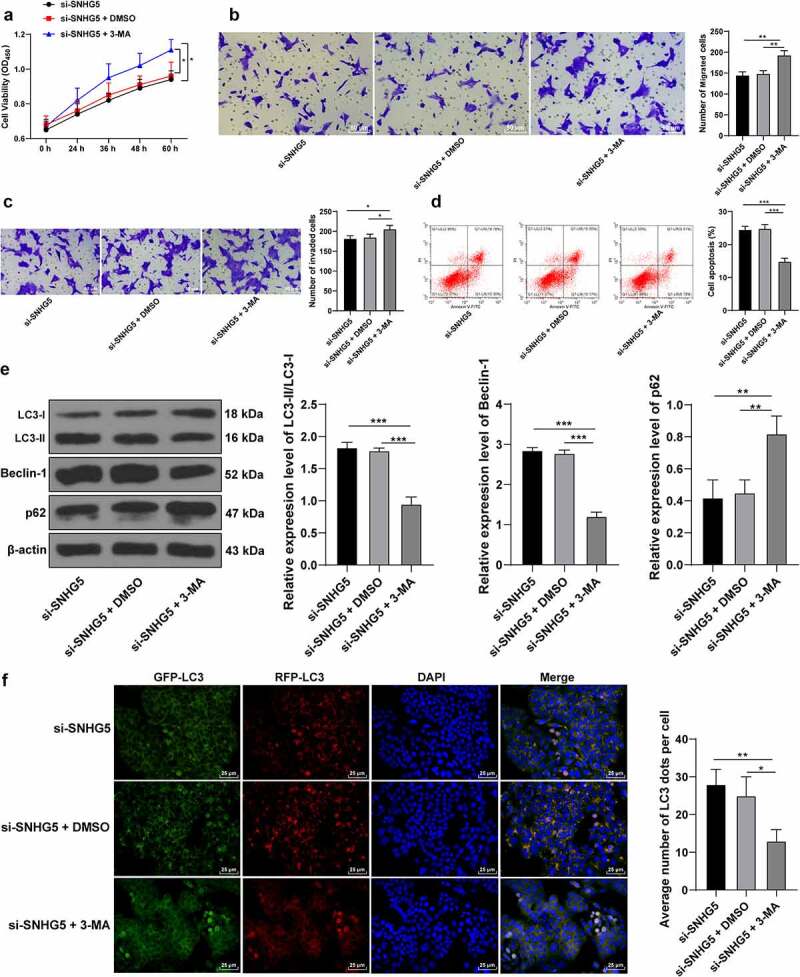
3-MA counteracts the promotive role of SNHG5 silencing in trophoblast autophagy. 3-MA was added into HTR-8/SVneo cells transfected with si-SNHG5, with DMSO addition as the control. (A) trophoblast proliferation was determined by CCK-8 method. (B) and (C) cell migration (b) and invasion (c) were measured by Transwell assays. (D) trophoblast apoptosis was assessed by flow cytometry. (E), levels of LC3, Beclin-1, and p62 were measured by Western blot analysis. (F) LC3 expression pattern was analyzed by RFP-GFP-LC3 tandem fluorescence assay. The independent experiments were performed 3 times. The results were presented as mean ± standard deviation. One-way ANOVA was used to analyze the data in panels (B) (C) (D) (E) and (F) and two-way ANOVA was used to analyze the data in panel A. Tukey’s multiple comparisons test was applied for the post hoc test. * *p* < 0.05, ** *p* < 0.01, *** *p* < 0.001.

To verify the mechanism of SNHG5 in PE by inhibiting trophoblast autophagy, an autophagy inhibitor 3-MA was supplemented in the HTR-8/SVneo cells along with downregulation of SNHG5, and the results revealed that 3-MA induced HTR-8/SVneo cell proliferation (*p* < 0.05, Figure 4a), and migration and invasion (*p* < 0.05, Figure 4b, c), while it inhibited HTR-8/SVneo cell apoptosis (*p* < 0.05, Figure 4d). The results of Western blot analysis revealed weakened LC3 and Beclin-1 expression pattern along with an elevated and p62 expression pattern upon 3-MA treatment (*p* < 0.05, Figure 4e). RFP-GFP-LC3 tandem fluorescence assay revealed that both the concentration of autophagosomes and autolysosomes were reduced in the HTR-8/SVneo cells upon 3-MA treatment (*p* < 0.05, figure 4f), thereby illustrating reduced autophagy in HTR-8/SVneo cells. These experimental results suggested that 3-MA annulled the promotive effect of SNHG5 silencing in trophoblast autophagy.

### SNHG5 competitively binds to miR-31-5p to facilitate SPARC transcription

**Figure 5.**
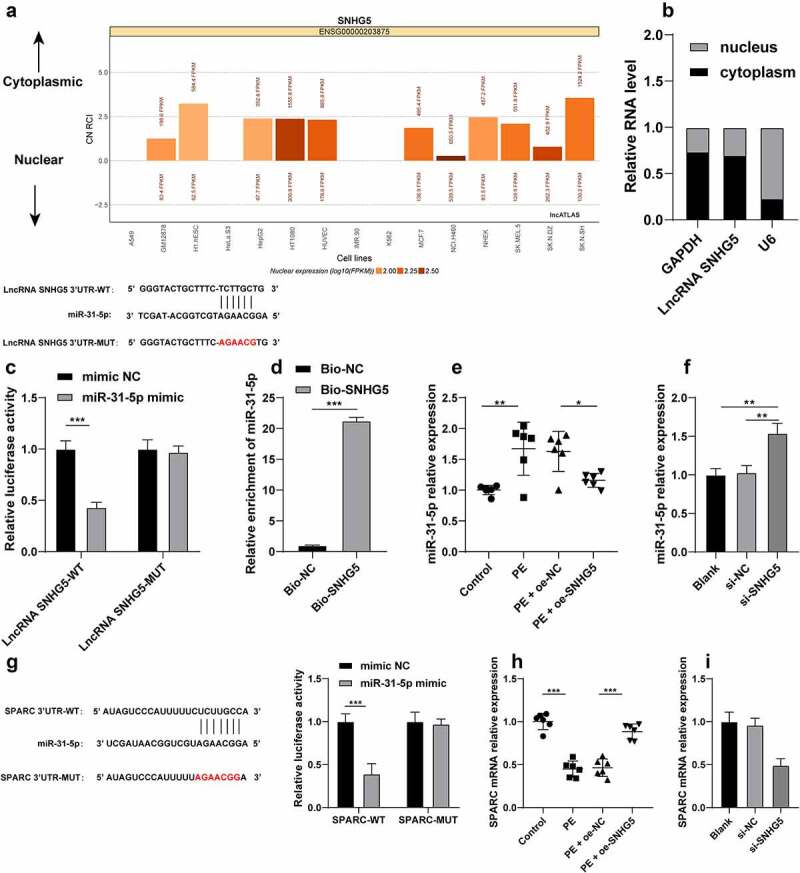
SNHG5 competitively binds to miR-31-5p to facilitate SPARC transcription. (A) SNHG5 subcellular localization was predicted by the LncATLAS website (http://lncatlas.crg.eu/). (B), SNHG5 subcellular localization in trophoblast was verified by fractionation of nuclear and cytoplasmic RNA. (C) and (D) the binding relation between SNHG5 and miR-31-5p was verified by dual-luciferase reporter gene assay (c) and RNA pull-down assay (d). (E) and (F), miR-31-5p expression pattern in mice (e) and trophoblasts (f) was detected by RT-qPCR. (G) the binding relation between miR-31-5p and SPARC was verified by dual-luciferase reporter gene assay. (H) and (I), SPARC expression pattern in mice (h) and trophoblasts (i) was detected by RT-qPCR. The independent experiments were performed 3 times. The results were presented as mean ± standard deviation. The *t*-test was used to analyze the data in panel D, one-way ANOVA was used to analyze the data in panels (E) (F) (H) and (I) and two-way ANOVA was used to analyze the data in panels (C) and (G). Tukey’s multiple comparisons test was applied for the post hoc test. * *p* < 0.05, ** *p* < 0.01, *** *p* < 0.001.

To extensively probe the molecular mechanism of SNHG5 in trophoblast autophagy, SNHG5 subcellular localization was predicted by the LncATLAS website (http://lncatlas.crg.eu/) and the result revealed principal localization of SNHG5 in the cytoplasm (Figure 5a). The result of fractionation of nuclear and cytoplasmic RNA verified a predominant expression of SNHG5 in the cytoplasm (Figure 5b), suggesting potential functionality of SNHG5 in PE via the ceRNA mechanism. Previously, miR-31-5p could improve trophoblast autophagy [15]. The RNA22 website (https://cm.jefferson.edu/rna22/Interactive/) predicted that SNHG5 can bind to miR-31-5p, and the combined results of the dual-luciferase reporter gene assay and RNA pull-down assay verified the ability of SNHG5 to bind to miR-31-5p (*p* < 0.05, Figure 5c-d). Additionally, the miR-31-5p expression pattern in each group of mice and cells was detected and the results revealed that miR-31-5p was overexpressed in the PE mice and HTR-8/SVneo cells. Our findings also elicited that SNHG5 overexpression could inhibit the miR-31-5p expression pattern in PE mice and SNHG5 silencing in HTR-8/SVneo cells increased miR-31-5p expression pattern (*p* < 0.05, Figure 5e-f). Moreover, the involvement of SPARC has been evident in autophagy [24] and PE pathogenesis [17]. The Targetscan website predicted SPARC as a downstream target gene of miR-31-5p, and dual-luciferase reporter gene assay showed that miR-31-5p overexpression inhibited the luciferase activity in the WT group, while no significant alterations were detected in the MUT group (*p* < 0.05, Figure 5g), indicating that miR-31-5p targeted SPARC 3’-UTR. Additionally, a poor expression pattern of SPARC was identified in the PE mice and HTR-8/SVneo cells, while it was increased in PE mice after SNHG5 overexpression (*p* < 0.05, Figure 5h-i) and reduced in the HTR-8/SVneo cells upon downregulation of SNHG5, thereby indicating that SNHG5 could improve SPARC transcription by competitively binding to miR-31-5p.

### miR-31-5p knockdown partially annuls the promotive role of SNHG5 silencing in trophoblast autophagy

**Figure 6.**
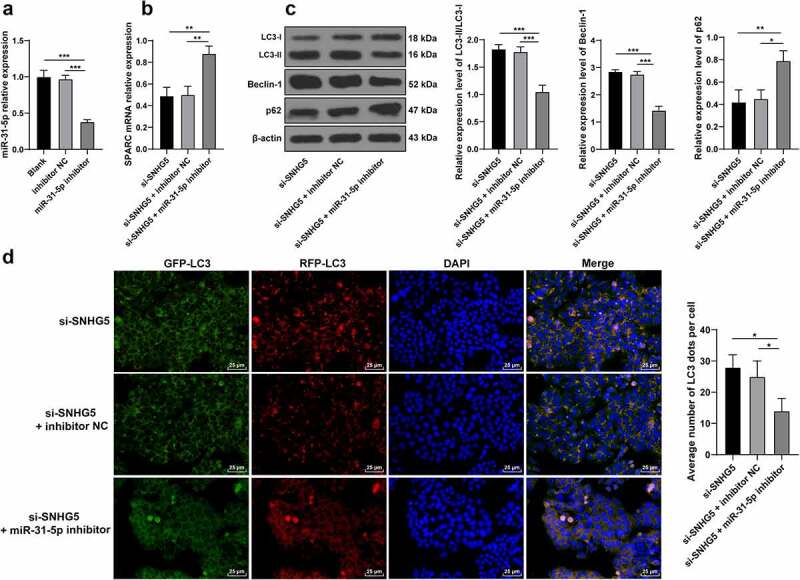
miR-31-5p knockdown partially annuls the promotive role of SNHG5 silencing in trophoblast autophagy. miR-31-5p inhibitor was transfected into HTR-8/SVneo cells, with inhibitor NC transfection as the control. (A) miR-31-5p inhibitor transfection efficiency was detected by RT-qPCR. (B) SPARC was determined by RT-qPCR. (C) levels of LC3, Beclin-1, and p62 were measured by Western blot analysis. (D) LC3 expression pattern in trophoblast was analyzed by RFP-GFP-LC3 tandem fluorescence assay. The independent experiments were performed 3 times. The results were presented as mean ± standard deviation. One-way ANOVA was used to analyze the data in panels (A) (B) (C) and (D). Tukey’s multiple comparisons test was applied for the post hoc test. * *p* < 0.05, ** *p* < 0.01, *** *p* < 0.001.

To verify whether SNHG5 could essentially regulate trophoblast autophagy by binding to miR-31-5p to upregulate SPARC, the miR-31-5p inhibitor or inhibitor NC was transfected into the HTR-8/SVneo cells (*p* < 0.05, Figure 6a), after which the degree of SPARC transcription in each group of trophoblasts was examined. The experimental results revealed improved SPARC transcription in the miR-31-5p inhibitor group (*p* < 0.05, Figure 6b). The protein levels of LC3, Beclin-1, and p62 in the trophoblasts of each group were detected and the findings revealed that the miR-31-5p inhibitor group reduced the ratio of LC3II/LC3l and Beclin-1 expression pattern, and elevated the p62 expression pattern (*p* < 0.05, Figure 6c). The results of RFP-GFP-LC3 tandem fluorescence assay revealed reduced concentrations of autophagosomes and autolysosomes in trophoblasts of the si-SNHG5 + miR-31-5p inhibitor group (*p* < 0.05, Figure 6d), thus eliciting that downregulation of miR-31-5p partially annulled the stimulative function of SNHG5 silencing in trophoblast autophagy.

## Discussion

PE is a complex disease during the gestation period and is responsible for the development of cardiovascular disorders and organ impairment that eventually lead to pregnancy termination [34]. PE has been frequently associated with elevated trophoblast autophagy level tissues [35]. LncRNA SNHG5 serves as a critical modulator of trophoblast function and can retard PE development [11]. In consistency with the aforementioned literature, the findings of our study further elucidated that lncRNA SNHG5 over-expression can inhibit trophoblast autophagy in PE *via* the miR-31-5p/SPARC axis.

Accumulating evidence has classified lncRNAs are vital indicators for PE detection and prognosis by manipulating trophoblast biological behaviors [36]. Previously, a weakened expression of lncRNA SNHG5 has been evident in intrauterine adhesions endometrium, which can facilitate the suppression of inflammatory responses and fibrinogen accumulation to reduce the degree of endometrial fibrosis and infertility [37]. An existing study implicated the downregulation of SNHG16 in PE tissues [38]. However, the definite role of SNHG5 in trophoblast autophagy in PE has not been elucidated. For a comprehensive analysis of the role of SNHG5 in trophoblast autophagy in PE, a PE mouse model was established, and a decreased expression pattern of SNHG5 was observed in PE mice, accompanied by an enhanced PE phenotype and tissue injury in PE mice, elevated proteinuria and blood pressure, increased TG, TC and LDL levels, decreased HDL level, elevated Bax level, and reduced Bcl-2 level. Autophagy, a fundamental mechanism to maintain cell homeostasis with a vital regulatory role in cell survival and death. Under normal conditions, autophagy is evident at a low level, with the elimination of damaged and defective organelles, misfolded proteins, and invading pathogens [39]. Autophagic cell death is integral in tumor inhibition and promotion, inflammation, and immune response [40,41]. Whether autophagy is harmful in its active form or its dysfunction depends on the specific disease and the autophagy level [42]. Autophagy dysregulation is implicated in several processes of pregnancy including embryogenesis, implantation, placenta formation, and delivery [43]. Accumulating research revealed that relative to the placenta of normal pregnancy, a considerably elevated level of autophagy activity was evident in PE placenta [21,44]. Autophagy could facilitate oxygen deprivation-induced trophoblast aging and subsequently exacerbate trophoblast dysfunction and deficiency [45]. With the termination of autophagy, the trophoblast viability was strengthened to facilitate placenta repair and PE mitigation [46]. Our results indicated that SNHG5 over-expression weakened trophoblast autophagy, as evidenced by the elevated LC3 level, reduced Beclin-1 level, limited conversion of LC3II to LC3l, and enhanced p62 level. In the *in vitro* experiments, the HTR-8/SVneo cells were transfected with si-SNHG5 to downregulate the SNHG5 expression, and our findings revealed that SNHG5 silencing improved trophoblast autophagy. Furthermore, we treated si-SNHG5-transfected HTR-8/SVneo cells with the autophagy inhibitor 3-MA, and our results revealed that 3-MA induced HTR-8/SVneo cell proliferation, migration, and invasion, but inhibited HTR-8/SVneo cell apoptosis. Moreover, our findings denoted that 3-MA treatment annulled the promotive effect of SNHG5 silencing on trophoblast autophagy, as evidenced by inhibited LC3 and Beclin-1 expressions, increased p62 expression, and reduced autophagosomes and autolysosomes. It was suggested that SNHG5 participated in the regulation of PE by inhibiting trophoblast autophagy.

Thereafter, we sought to validate the downstream mechanism of SNHG5. We predicted the subcellular localization of SNHG5 and validated the localization of SNHG5 in the cytoplasm, thus indicating that SNHG5 could potentially modulate PE progression via the ceRNA network. In the current study, dual-luciferase reporter gene assay and RNA pull-down assay revealed that SNHG5 could competitively bind to miR-31-5p. Additionally, an elevated miR-31-5p expression was identified in PE, where it impeded cell proliferation, mobility, and angiogenesis to exacerbate the degree of PE [47]. Moreover, miR-31-5p exacerbated PE by increasing hypertension, endothelial impairment, and trophoblast loss [48]. To verify the function of miR-31-5p in trophoblast autophagy in PE, the miR-31-5p inhibitor was transfected into the HTR-8/SVneo cells and it was identified that downregulation of miR-31-5p suppressed trophoblast autophagy. Consistently, an existing study classified that miR-31-5p as the causative factor for abolished trophoblast migration and augmented trophoblast autophagy [16]. Altogether, these findings implicated miR-31-5p as a potential risk factor in trophoblast autophagy in PE. Thereafter, the downstream targets of miR-31-5p were predicted through the database, from which SPARC was chosen as the research subject. An existing study demonstrated that SPARC improved trophoblast mobility and modulated the function of the placenta, uterus, and ovary [18]. SPARC induces phenotypic modulation of human brain vascular smooth muscle cells via AMPK/mTOR-mediated autophagy, as shown by the variances of LC3 and Beclin-1 expression and p62 self-renewal [24]. However, the role of SPARC in trophoblast autophagy in PE remains elusive. Our findings verified that miR-31-5p can target SPARC, with a poor expression of SPARC in PE mice and HTR-8/SVneo cells. Transfection of miR-31-5p inhibitor increased the SPARC transcription level in HTR-8/SVneo cells. An existing study illustrated the role of the lncRNA DANCR/miR-214-5p/PI3K/AKT axis in HRT-8/Svneo cell proliferation, migration, and invasion [49]. This study is the first to report that SNHG5 can influence trophoblast migration, invasion, apoptosis, and autophagy in PE via manipulation of the miR-31-5p/SPARC axis.

## Conclusion

To conclude, our findings elicited the functionality of SNHG5 as a ceRNA to competitively bind to miR-31-5p and upregulate SPARC transcription, thus inhibiting trophoblast autophagy in PE. However, the current study was restricted due to the following limitations: firstly, we only studied the PE mouse model induced by injection of TLR9 agonist, and our findings need further validation in other PE mouse models. Secondly, the HTR-8/SVneo cells were chosen for cell experiments. HTR-8/SVneo cells have extensive application in the modeling of extravillous trophoblast cells, but the data from this cell line cannot be used for primary trophoblast cells. Therefore, the application of choriocarcinoma cell line (such as BeWo and JEG-3) or isolating primary placental villous trophoblast is warranted for further exploration. Thirdly, we did not harvest the placental tissues of PE patients for detection. Our future studies will verify the underlying mechanism of SNHG5/miR-31-5p/SPARC in the placental tissues of PE patients and human primary villous trophoblasts.

## Data Availability

The data that support this study are available from the corresponding author upon reasonable request.
